# Supplementary material for: Insulin‐Dependence and Survival in Pancreatic Neuroendocrine Tumors: Results From the US‐NTSG Group
Source: J Surg Oncol. 2026 Jan 11;133(2):153–60. doi: 10.1002/jso.70174 (PMC12863235; doi:10.1002/jso.70174)
Supplement: Supplementary file 1 — Supplementary Tables. [file JSO-133-153-s001.docx]

| **Supplementary Table 1.** Pre-operative IDDM-related Comorbidities by Insulin-dependence | | | | | | |
| --- | --- | --- | --- | --- | --- | --- |
| **Variable** | | **No DM,**  **N (%)** | **NIDDM**  **N (%)** | **IDDM**  **N (%)** | ***P*-value** | |
| **Total** | | **870** | **168** | **84** | ***All*** | ***IDDM vs***  ***NIDDM + No DM*** |
| Hypertension | Yes | 317 (36.4) | 110 (65.5) | 53 (63.1) | **<0.001** | **<0.001** |
|  | No | 534 (61.4) | 57 (33.9) | 31 (36.9) |  |  |
|  | *Unknown* | 19 (2.2) | 1 (0.6) | 0 (0.0) |  |  |
| Previous Coronary Event | Yes | 48 (5.5) | 13 (7.7) | 14 (16.7) | **0.001** | **0.002** |
|  | No | 801 (92.1) | 154 (91.7) | 70 (83.3) |  |  |
|  | *Unknown* | 21 (2.4) | 1 (0.6) | 0 (0.0) |  |  |
| Chronic Heart Failure | Yes | 7 (0.8) | 1 (0.6) | 2 (2.4) | 0.245 | 0.126 |
|  | No | 845 (97.1) | 166 (98.8) | 82 (97.6) |  |  |
|  | *Unknown* | 18 (2.1) | 1 (0.6) | 0 (0.0) |  |  |
| Dyspnea | Yes | 16 (1.8) | 3 (1.8) | 4 (4.8) | **0.176** | 0.087 |
|  | No | 835 (96.0) | 164 (97.6) | 80 (95.2) |  |  |
|  | *Unknown* | 19 (2.2) | 1 (0.6) | 0 (0.0) |  |  |
| Acute Renal Failure | Yes | 0 (0.0) | 1 (0.6) | 0 (0.0) | 0.121 | 0.436 |
|  | No | 852 (97.9) | 166 (98.8) | 84 (100) |  |  |
|  | *Unknown* | 18 (2.1) | 1 (0.6) | 0 (0.0) |  |  |
| Chronic Renal Failure | Yes | 7 (0.8) | 1 (0.6) | 0 (0.0) | 0.053 | 0.126 |
|  | No | 845 (97.1) | 166 (98.8) | 83 (98.8) |  |  |
|  | *Unknown* | 18 (2.1) | 1 (0.6) | 1 (1.2) |  |  |
| Ascites | Yes | 1 (0.1) | 1 (0.6) | 1 (1.2) | 0.091 | 0.133 |
|  | No | 851 (97.8) | 166 (98.8) | 83 (98.8) |  |  |
|  | *Unknown* | 18 (2.1) | 1 (0.6) | 0 (0.0) |  |  |
| Other Malignancy | Yes | 37 (4.3) | 15 (8.9) | 3 (3.6) | 0.072 | 0.595 |
|  | No | 816 (93.8) | 152 (90.5) | 81 (96.4) |  |  |
|  | *Unknown* | 17 (2.0) | 1 (0.6) | 0 (0.0) |  |  |
| Disseminated Cancer | Yes | 57 (6.6) | 15 (8.9) | 10 (11.9) | 0.120 | 0.145 |
|  | No | 795 (91.4) | 152 (90.5) | 74 (88.1) |  |  |
|  | *Unknown* | 18 (2.1) | 1 (0.6) | 0 (0.0) |  |  |
| Identified Genetic Mutation | Yes | 89 (10.2) | 12 (7.1) | 11 (13.1) | 0.063 | 0.382 |
|  | No | 764 (87.8) | 155 (92.3) | 73 (86.9) |  |  |
|  | *Unknown* | 17 (2.0) | 1 (0.6) | 0 (0.0) |  |  |
| Pancreatitis | Yes | 43 (4.9) | 13 (7.7) | 9 (10.7) | 0.063 | 0.076 |
|  | No | 808 (92.9) | 154 (91.7) | 75 (89.3) |  |  |
|  | *Unknown* | 19 (2.2) | 1 (0.6) | 0 (0.0) |  |  |
| Anemia | Yes | 63 (7.2) | 16 (9.5) | 8 (9.52%) | 0.249 | 0.449 |
|  | No | 787 (90.5) | 151 (89.9) | 76 (90.5%) |  |  |
|  | *Unknown* | 20 (2.3) | 1 (0.6) | 0 (0.00%) |  |  |

**Supplementary Table 2.** Cancer-specific Survival Cox Univariate Analysis

| **Variable** | | | **HR** | **95% CI** | **p-value** |
| --- | --- | --- | --- | --- | --- |
| **DM** | **IDDM** | | **2.65** | **1.41 – 4.99** | **0.003** |
|  | NIDDM | | 0.86 | 0.41 – 1.81 | 0.685 |
| **Male** | | | 0.92 | 0.57 – 1.47 | 0.713 |
| **Age** | | | 1.01 | 0.99 – 1.03 | 0.475 |
| **BMI** | | | 0.95 | 0.90 – 0.99 | **0.028** |
| **ASA Class** | | III | 0.93 | 0.54 – 1.60 | 0.793 |
|  |  | **IV** | **4.53** | **1.84 – 11.1** | **<0.001** |
| **HbA1c (n=167)** | | | 1.41 | 0.90 – 2.20 | 0.134 |
| **Serum Glucose** | | | 1.00 | 0.99 – 1.01 | 0.284 |
| **PNET Location: Head/Neck** | | | 1.14 | 0.71 – 1.85 | 0.586 |
| **Hypertension** | | | 1.03 | 0.63 – 1.70 | 0.904 |
| **Previous Coronary Event** | | | 0.56 | 0.13 – 2.31 | 0.427 |
| **Dyspnea** | | | 1.07 | 0.15 – 7.71 | 0.950 |
| Note:  *HR* Hazard Ratio; *CI* Confidence Interval; *DM* diabetes mellitus; *IDDM* Insulin dependent diabetes mellitus; *NIDDM* non-insulin dependent diabetes mellitus; *HbA1c* glycosylated hemoglobin A1c | | | | | |
